# Health disparity and COVID‐19—A retrospective analysis

**DOI:** 10.1002/hsr2.345

**Published:** 2021-08-05

**Authors:** Sanjay Sarkar, Archie Taylor, Pratik Dutta, Meghna Bajaj, Justin Nash, Martha Ravola, Sofia Ievleva, Cardarius Llyod, Praise Ola, Brenita Jenkins, Bidisha Sengupta, Debarshi Roy

**Affiliations:** ^1^ Department of Genetics University of North Carolina – Chapel Hill Chapel Hill North Carolina USA; ^2^ School of Nursing Alcorn State University Lorman Mississippi USA; ^3^ Indian Institute of Technology – Patna Patna India; ^4^ Department of Chemistry and Physics Alcorn State University Lorman Mississippi USA; ^5^ Department of Biology Alcorn State University Lorman Mississippi USA; ^6^ Department of Human Science Alcorn State University Lorman Mississippi USA; ^7^ Department of Chemistry and Biochemistry Stephen F Austin State University Nacogdoches Texas USA

**Keywords:** COVID‐19, disparity, Mississippi, obesity, SARS‐CoV2

## Abstract

**Background and Aims:**

According to the World Health Organization (WHO), more than 75.7 million confirmed cases of coronavirus disease 2019 (COVID‐19), a global pandemic caused by severe acute respiratory syndrome coronavirus 2 **(**SARS‐CoV‐2), have been reported so far. Researchers are working relentlessly to find effective solutions to this catastrophe, using genomic sequence‐based investigation, immunological analysis, and more. The role of health disparity has also emerged as an intriguing factor that made a huge impact on the lives of people.

**Methods:**

We analyzed various factors that triggered the health disparity in the United States of America along with the rate of COVID‐19 morbidity and mortality. Furthermore, we have also focused on the State of Mississippi, which is suffering from an extreme health disparity. Data have been obtained from publicly available data sources including, Center for Disease Control and Prevention and Mississippi State Department of Health. Correlation analysis of the dataset has been performed using R software.

**Results:**

Our analysis suggested that the COVID‐19 infection rate per 100 000 people is directly correlated with the increasing number of the African American population in the United States. We have found a strong correlation between the obesity and the COVID‐19 cases as well. All the counties in Mississippi demonstrate a strong correlation between a higher number of African American population to COVID‐19 cases and obesity. Our data also indicate that a higher number of African American populations are facing socioeconomic disadvantages, which enhance their chances of becoming vulnerable to pre‐existing ailments such as obesity, type‐2 diabetes, and cardiovascular diseases.

**Conclusion:**

We proposed a possible explanation of increased COVID‐19 infectivity in the African American population in the United States. This work has highlighted the intriguing factors that increased the health disparity at the time of the COVID‐19 pandemic.

**What is known about this topic**

- There has been significant variation in the severity (morbidity and mortality) of the coronavirus disease 2019 (COVID‐19) disease caused by severe acute respiratory syndrome coronavirus 2 (SARS‐CoV‐2) among people from diverse background.
- Few reports have suggested that incidences of COVID‐19 cases, morbidity, and mortality among African American people are higher compared to white American people.
- Health disparity has been suggested to be one of the many factors responsible for the wide variation in the incidences as well as sufferings caused by COVID‐19 in different population in the United States.

**What this paper adds**

- Our analyses show that various socioeconomic factors have resulted in health disparity among different population in the United States resulting in increased sufferings due to COVID‐19 in African American.
- There is significant positive correlation among the number of COVID‐19 cases and the total number of the African American population in the United States.
- Our analyses also suggested that in the state of Mississippi, a significant disparity between white and black American people in different socioeconomic factors is present, and this resulted in health disparity, which positively correlated to the COVID‐19 disease severity.

## 1 INTRODUCTION

The world is currently under threat due to an unprecedented pandemic situation, the novel coronavirus disease 2019 (COVID‐19) that originally started at Wuhan, China, in December 2019 and since then, has very rapidly spread to different parts around the globe causing about 75.7 million confirmed cases of COVID‐19 and 1 690 061 deaths from 214 countries and territories.^1^ The disease is caused by a coronavirus, known as severe acute respiratory syndrome coronavirus 2 (SARS‐CoV‐2), which has a positive‐sense single‐stranded RNA genome.^2^ Although the virus can infect humans of all ages, sex, and ethnicities, the magnitude of suffering may vary greatly among the population depending on the age groups, sex, ethnicities, and comorbidities.^3^ The SARS‐CoV‐2‐infected individuals can either remain asymptomatic or may show a range of symptoms including but not limited to common cold such as fever, chills, muscle pain, fatigue, loss of taste or smell, vomiting, diarrhea, to more severe diseases like bronchitis, pneumonia, severe acute respiratory distress syndrome (ARDS), and multiorgan failure leading to death.^4^ The mortality rate in COVID‐19 diseases is around 6.3% in the United States (per CDC) although it varies depending upon the human population and the geographical regions. SARS‐CoV‐2 is primarily transmitted via the respiratory route by inhalation of respiratory droplets.^5^ The incubation period of SARS‐CoV‐2 varies from 2 to 14 days with a median of 4‐5 days. The virus binds to the cells of the upper respiratory tract expressing the entry receptor, angiotensin‐converting enzyme 2, and the protease,TMPRSS2.^2^, ^6^ On entering the cells, the virus multiplies utilizing the host cell machinery, releasing the progeny virions resulting in pyroptosis of the host cells.^7^ These, in turn, cause the release of damage‐associated molecular patterns including adenosine triphoshate, nucleic acids, and Apoptosis‐associated speck‐like protein containing a C‐terminal caspase recruitment domain oligomers, which are recognized by the adjacent epithelial cells, endothelial cells, and alveolar macrophages.^7^ This leads to the expression of proinflammatory cytokines and chemokines including IL‐6, IP‐10, and macrophage inflammatory protein 1α (MIP1α), which attracts monocytes, macrophages, and T‐cells to the site of inflammation further escalating inflammation and inflammatory mediators.^7^, ^8^ In the absence of a protective antiviral immune response, this overproduction of proinflammatory cytokines results in the tissue destruction of the lung leading to ARDS.^9^ The proinflammatory cytokines reach other organs, causing tissue destruction eventually resulting in the multiorgan failure leading to the death of the infected individual.

In the United States, COVID‐19 has made a huge impact on the lives and economy of people. To date, almost 15 million people are reported to be COVID‐19 positive in the United States out of which 0.3 million people have died from this^4^ (data accessed on December 7, 2020). Although the recovery rate is currently shown to be promising, but the severity of COVID‐19 infection can also be dependent on the pre‐existing health condition of the individual.^10^ Reports have strongly suggested that pre‐existing conditions are playing a crucial role in the devastating statistics of COVID‐19 disease.^10^, ^11^ People affected with cardiovascular diseases such as ischemia, cardiac failure, etc., are found to have a poorer prognosis when got infected by the SARS‐CoV‐2 compared to the healthy individual.^12^ It is also reported that type‐2 diabetic patients suffering from high blood glucose levels and poorer immunity are affected heavily by this deadly virus.^10^ Obese people are threatened by COVID‐19 due to a weak immunity as well.^13^

In the United States, people of color (mainly African American and Hispanics/Latinos) are generally tending to have a poor health index with decreasing socioeconomic status.^14^, ^15^ The challenges faced by the people of color are much more prominent compared to white Caucasians during any health crisis such as COVID‐19 pandemic in the United States.^14^, ^15^, ^16^ Epidemiological studies reveal that African Americans are more prone to develop hypertension, cardiac diseases, obesity, and diabetes in the span of their life compared to a white American.^17^, ^18^, ^19^, ^20^ States having a higher number of African American populations in the United States are facing severe health disparity.^15^ For example, the state of Mississippi, which has the maximum percentage of the African American population (37% African American population according to census.gov) in the United States, is facing a severe health disparity (2009).^21^ Mississippi has a 40% obesity rate in African Americans compared to 35% of the white population. The racial or ethnic differences that lie within the health sector are the determinants of health disparity.^22^ Socioeconomic factors such as health insurance, employment, educational attainment, etc., are also considered as triggering factors to determine health disparity in any population.

Our current research emphasizes the various socioeconomic and health parameters, which are the significant drivers of health disparity in the United States. We analyzed if these parameters are triggering COVID‐19 in the United States as well. The overall goal of our study is to identify if there is any significant association between health disparity parameters and COVID‐19 disease in different ethnic population in the United States with a special emphasis on Mississippi.

## 2 MATERIALS AND METHOD

### 2.1 Source

Statistical data related to COVID‐19 cases and deaths in different states of the United States were found from the CDC website (covid.cdc.gov).

Data related to COVID‐19 cases and deaths in Mississippi were collected (28 October 2020) from the Mississippi State Department of Health website. (https://msdh.ms.gov/msdhsite/_static/14,0,420.html#county)

Different health parameters for the state of Mississippi were also collected from the Mississippi State Department of Health website.

Data for socioeconomic parameters were available from source livestories.com and data.census.gov.

### 2.2 Data analysis

Initial steps of analysis included organizing and summarizing the collected data from different data sources.

1. Correlation. This calculation is important to enumerate the association between the different variables by calculating the Pearson correlation between all pairs of variables and their significance test. Correlation coefficient calculations were carried out by R software. *P*‐values were set at .05 and less than .05 were considered significant. The color‐coded square boxes indicate statistical significance; white boxes represent no significance.
2. We have applied the clustering algorithm to the Mississippi population. This clustering algorithm group and the rank of the population based on the average ratio of the black and white population, income, COVID‐19 infected, and COVID‐19 death. Here, we have used K‐means clustering for performing this grouping. We have applied the k‐means clustering technique on the Mississippi state data. The data contain the information of all 82 countries of Mississippi state. For each country, we have black and white population ratio, COVID‐19 cases ratio, COVID‐19 deaths ratio, income ratio, and the insurance ratio. Along with these pieces of information, for each country, we have the diabetic and obesity percentage of the population. To grouping the countries into several clusters, we have applied the K‐means clustering algorithm to this dataset. The number of the optimal clusters is decided by using the Elbow method and the Silhouette score. The obtained clustering result is shown using the colored map.

## 3 RESULT

COVID‐19 infection has been widespread all over the world and affected almost all nations. In the United States, the infectivity of COVID‐19 was unequally distributed all over the 50 different states. The minority population is found to be severely affected by COVID‐19 infection in the United States.^14^ Pre‐existing conditions, such as obesity, cardiovascular diseases, and type‐2 diabetes, are suggested as crucial factors for triggering severe form of COVID‐19 disease.^3^ We were curious to analyze if the percentage of the African American population, rate of pre‐existing conditions, etc., are important for the unequal distribution of COVID‐19 infections, disease severity, and COVID‐19‐related deaths in the United States.

**FIGURE 1:**
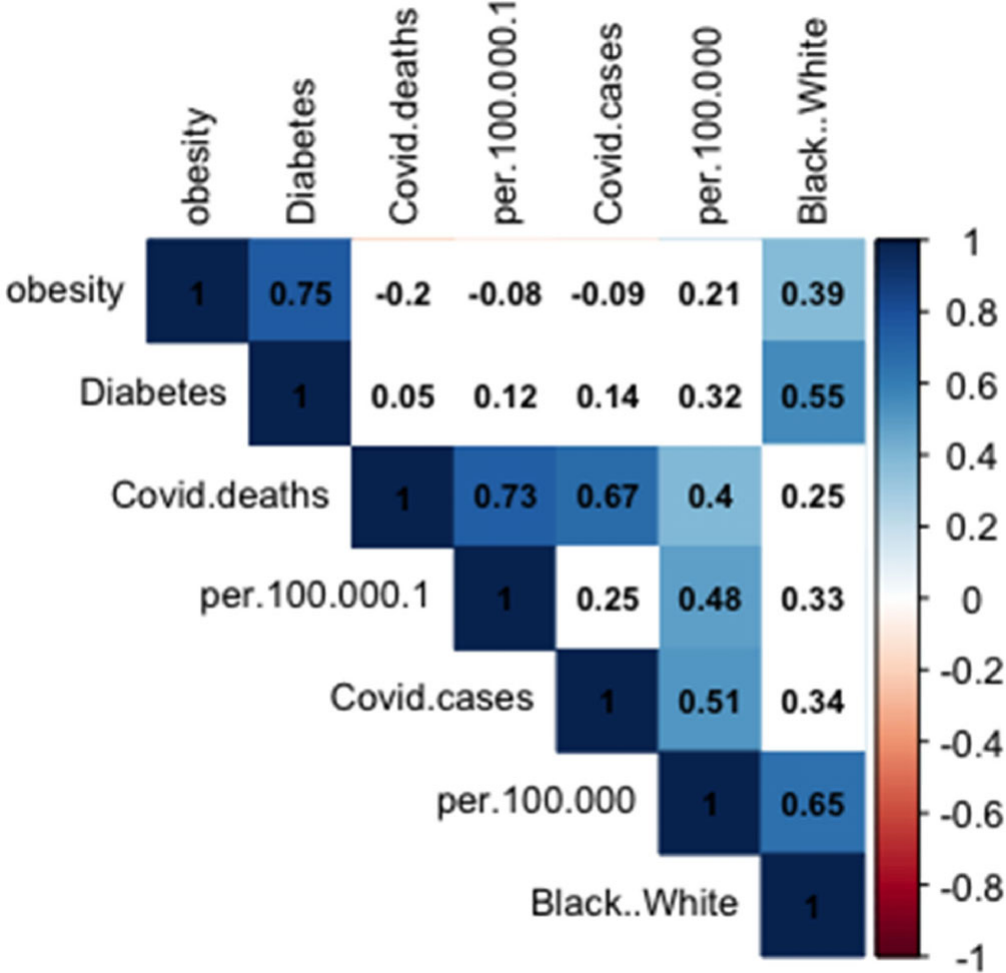
Analysis of pre‐existing health conditions and coronavirus disease 2019 (COVID‐19) infection in US states

We have calculated Pearson's correlation coefficient on the following parameters: the ratio of African American or black (B) and Caucasian or white (W) population in 50 US states; COVID‐19 incidences per 100 000 population in each state; COVID‐19‐related deaths per 100 000 population in each state; the percentage of obese population in each state; and the percentage of type‐2 diabetic population in each state. Our results are summarized in Figure 1. The correlation coefficients are ranged from −1 to +1 where values close to +1 represent the positive and stronger correlation. Although we have not found any correlation between the COVID‐19‐related deaths and B:W population ratio in the US states, but a positive and significant (*P* < .01) strong correlation between the number of COVID‐19 cases and B:W population is found (*r* = 0.65). Our analyses strongly indicate a significant (*P* < .01) positive correlation between obesity and type‐2 diabetes incidences to B:W population in the US states (*r* = 0.39, 0.55). An association between the increased black population to metabolic diseases is observed throughout the US states; this trend indicates an increasing health disparity and inequality in the United States. The growing number of metabolic diseases is also considered as the major pre‐existing conditions, which could accelerate the COVID‐19 infection rate. The black population in the United States are already suffering from high percentage of obesity and diabetes, which may result in increased incidences of COVID‐19 infection in certain states where there is more black population.^10^ Incidences of COVID‐19 are reported higher among the African American population, which is also reflected from these data.

**FIGURE 2:**
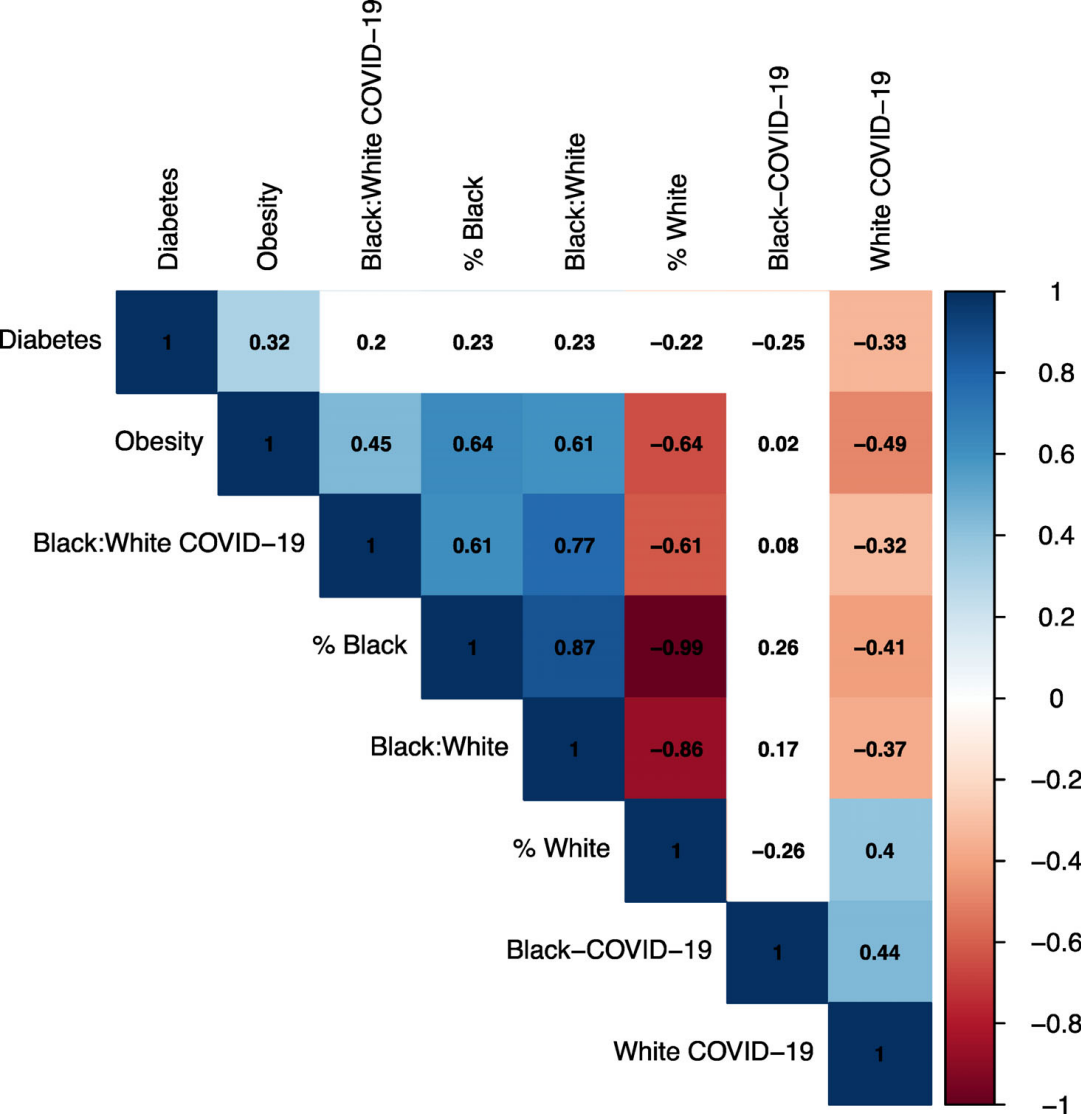
Analysis of pre‐existing health conditions and coronavirus disease 2019 (COVID‐19) infection in Mississippi counties

Mississippi is one of the southern states of the United States, which has the highest African American population (37%) as of 2020. This state is currently ranked as the most obese state (40.8% adult obesity, https://www.cdc.gov/obesity/data/prevalence-maps.html) in the United States and ranked as the second highest in the incidence of type‐2 diabetes.^23^ The African American population in this state is largely affected during the pandemic. Our effort was to analyze the impact of COVID‐19 in each of the 82 counties in this state and examine any association between COVID‐19 infection and death rate to the racial disparity and health disparity parameters. Figure 2 summarizes the correlation between the different parameters. Analysis of correlation coefficient suggests a positive correlation between obesity to increased COVID‐19 cases in the black population (*r* = 0.45). The ratio between B:W population is also positively correlated with the percent of obese population in Mississippi. Overall, these data indicate that there is an increased obese population among the African Americans in Mississippi, which are also vulnerable toward COVID‐19 infection compared to the white population.

**FIGURE 3:**
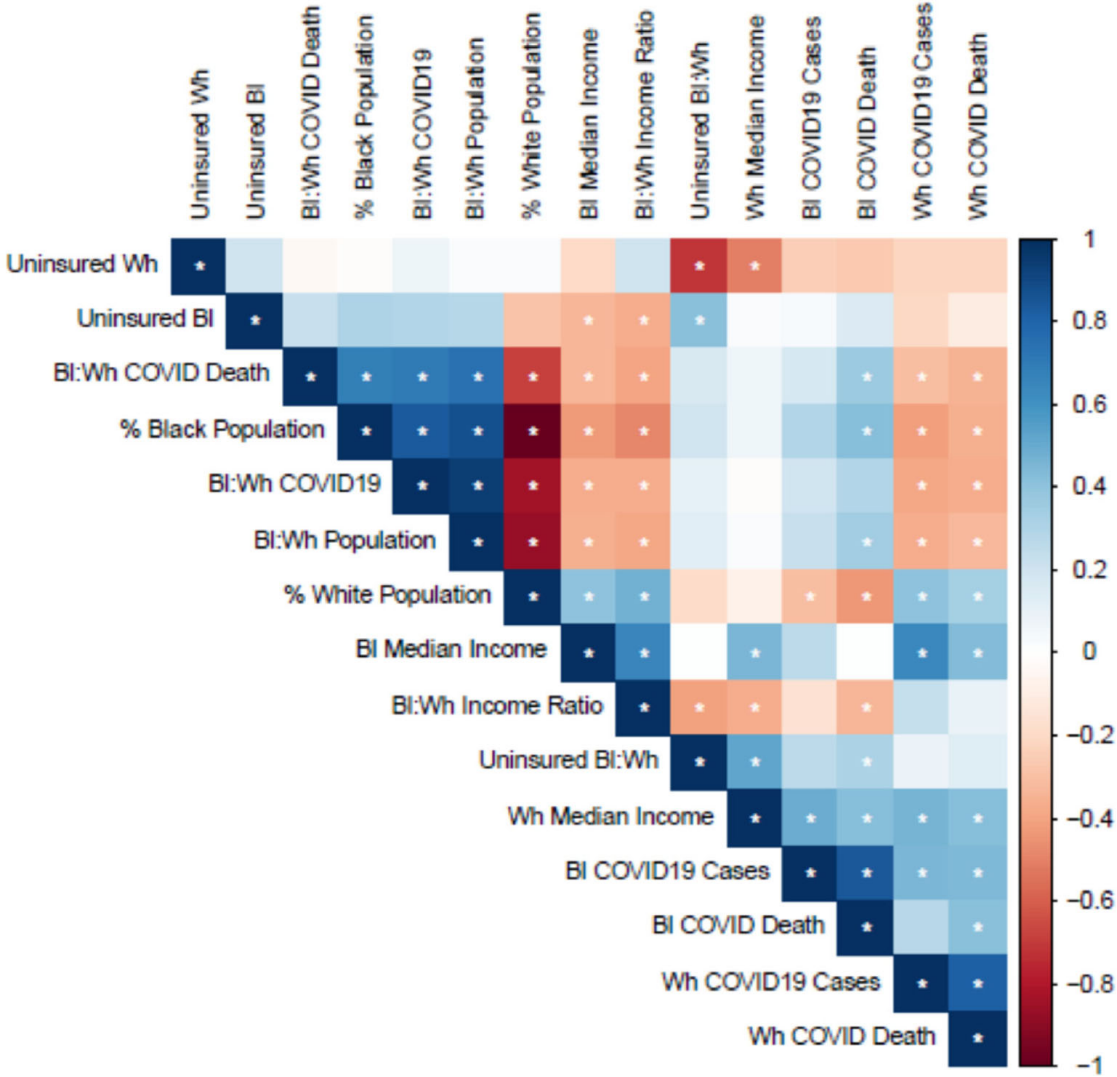
Analysis of socioeconomic parameters and coronavirus disease 2019 (COVID‐19) infection in Mississippi Counties. Bl = black, Wh = white. Asterisk (*) indicates *P*‐value smaller than .05

Next, we have analyzed the correlation between the socioeconomic parameters to the COVID‐19 cases and deaths in Mississippi counties (Figure 3). We have analyzed the median income and percentage of uninsured black and white population in 82 different counties. We find a negative correlation between black:white median income ratio to COVID‐19‐mediated death (−0.33) and cases (−0.36) in the black population. These data indicate that higher median income being a crucial socioeconomic factor that can eliminate the devastating impact of COVID‐19 in people of color. Next, we have analyzed the impact of health insurance on the COVID‐19‐infected population. We did not see a strong correlation between the percentage of the uninsured black population to COVID‐19 cases or deaths, but we found a positive correlation between uninsured black: white ratio to black COVID‐19 cases (0.32), which indicate that the lack of health insurance may be pivotal for the increasing COVID‐19 cases and deaths in African American people in Mississippi. A more detailed study (eg, performing sample study analysis from healthcare providers, from county health officials, etc.) is required to find the exact association between health insurance, pre‐existing diseases, and COVID‐19 cases.

**FIGURE 4:**
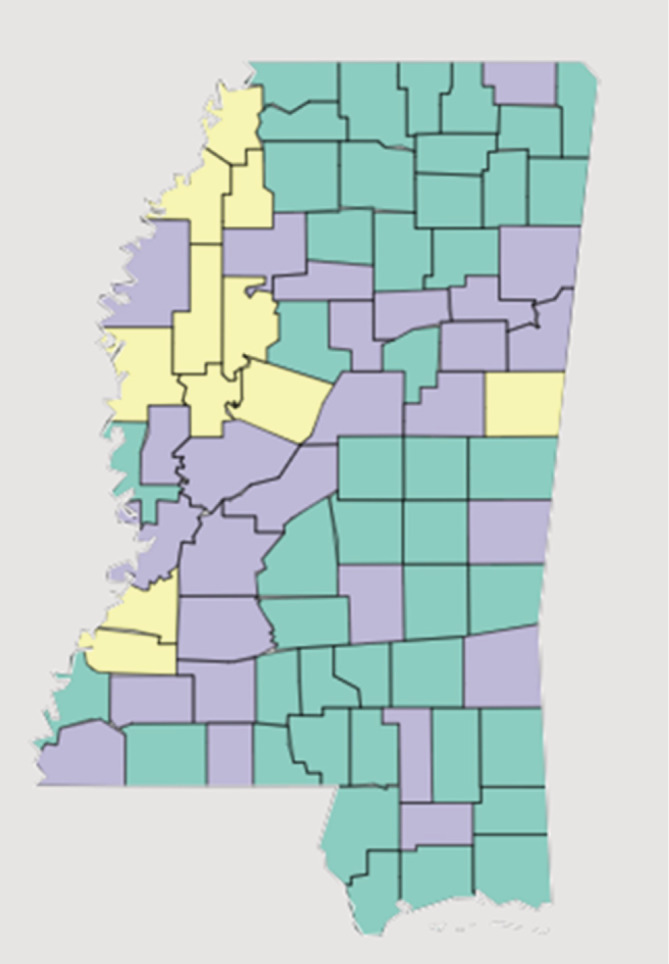
Mississippi clusters based on health disparity in different counties. The clusters are designed on the basis of pre‐existing health conditions and socioeconomic parameters. cluster_1 (green, 44 countries): [“Adams,” “Amite,” “Benton,” “Calhoun,” “Carroll,” “Chickasaw,” “Choctaw,” “Clarke,” “Covington,” “Desoto,” “George,” “Greene,” “Hancock,” “Harrison,” “Issaquena,” “Itawamba,” “Jackson,” “Jasper,” “Jefferson Davis,” “Jones,” “Kemper,” “Lafayette,” “Lamar,” “Lawrence,” “Leake,” “Lee,” “Marion,” “Marshall,” “Neshoba,” “Newton,” “Panola,” “Pearl River,” “Perry,” “Pontotoc,” “Prentiss,” “Rankin,” “Scott,” “Simpson,” “Tate,” “Tippah,” “Tishomingo,” “Union,” “Walthall,” and “Yalobusha”] cluster_2 (yellow, 11 countries): [“Claiborne,” “Coahoma,” “Holmes,” “Humphreys,” “Jefferson,” “Leflore,” “Noxubee,” “Quitman,” “Sunflower,” “Tunica,” and “Washington”] cluster_3 (gray, 27 countries): [“Alcorn,” “Attala,” “Bolivar,” “Clay,” “Copiah,” “Forrest,” “Franklin,” “Grenada,” “Hinds,” “Lauderdale,” “Lincoln,” “Lowndes,” “Madison,” “Monroe,” “Montgomery,” “Oktibbeha,” “Pike,” “Sharkey,” “Smith,” “Stone,” “Talahatchie,” “Warren,” “Wayne,” “Webster,” “Wilkinson,” “Winston,” and “Yazoo”]

The clustering algorithm (Figure 4) gives a set of clusters. The clusters are then ranked based on the disparity or differences of the counties. The clusters are designed on the basis of pre‐existing health conditions and socioeconomic parameters. K‐means clustering produced the three clusters which are *Cluster 1* containing 44 counties where the counties belonging to this cluster show higher insurance ratio and a higher percentage of diabetic patients. *Cluster‐2* contains 11 counties where the counties belonging to this cluster show higher COVID‐19 deaths ratio (B/W) and the population ratio (B/W). *Cluster‐3* contains 27 counties where the counties belonging to this cluster show a high value of insurance ratio. These data are useful to distinguish the counties based on different socioeconomic and health parameters, which are crucial determinants of health disparity and COVID‐19 infection in the state of Mississippi.

**FIGURE 5:**
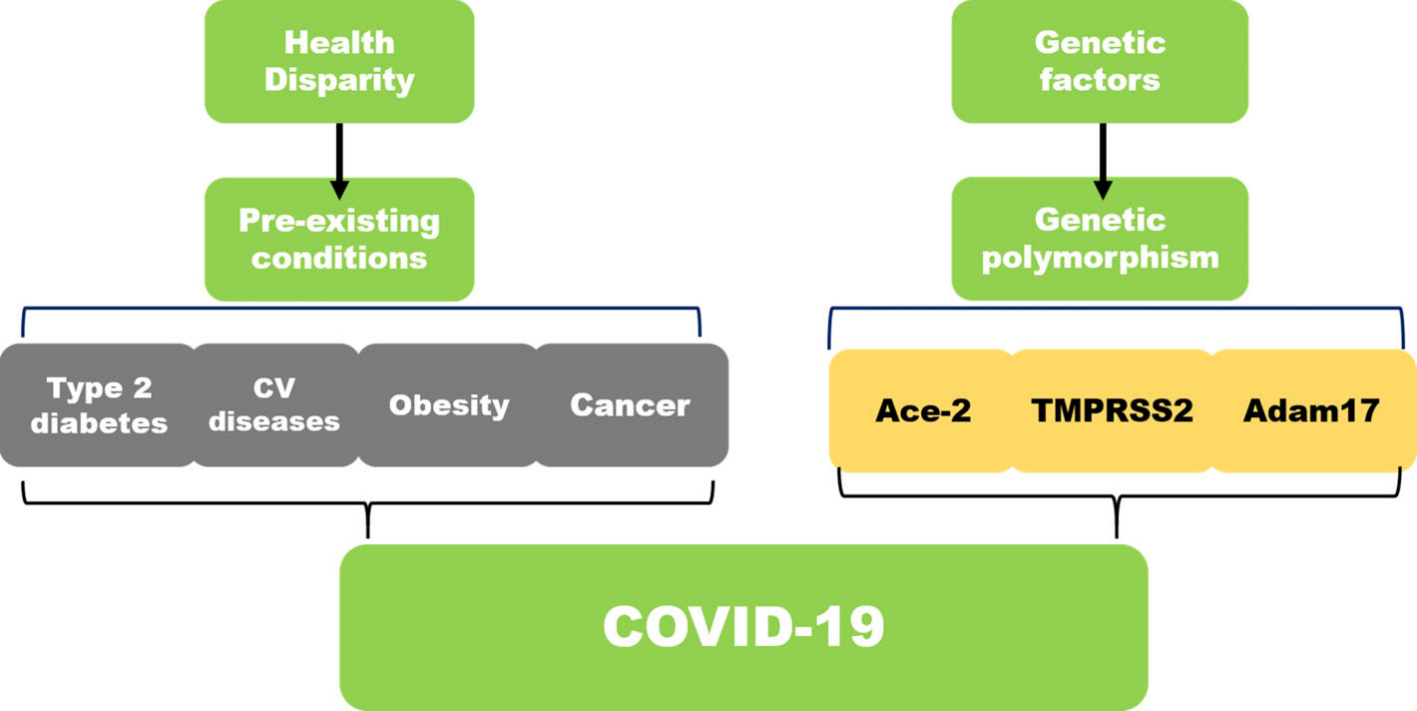
Coronavirus disease 2019 (COVID‐19) infection and the associated factors. A schematic depicting the various factors promoting COVID‐19 infection. CVD, cardiovascular

Figure 5 narrates the finding from our analysis with other existing factors, which may contribute to COVID‐19 infection in the general population. This schematic diagram highlights the pre‐existing conditions health disparity and the genetic factors, which are impacting the occurrence of COVID‐19 infection. Several different polymorphisms in ACE‐2, TMPRSS2, and Adam17 genes are now found related to COVID‐19 infections.^24^ The pre‐existing diseases including cardiovascular diseases, obesity, and type‐2 diabetes are among the major ailments, which are studied in accordance with COVID‐19 infection.^25^, ^26^, ^27^ Data also suggest that cancer could play an important role in triggering the severity of COVID‐19 due to a lowered immune response in cancer patients due to chemotherapy‐related treatments.^10^, ^28^

## 4 DISCUSSION

In this study, we found that health disparity and socioeconomic disparities found within the communities of color in the United States significantly make these population prone to many chronic pre‐existing conditions such as type‐2 diabetes, obesity, and cardiovascular diseases, which in turn enhance the COVID‐19 disease prevalence as well as severity. We analyzed the association between health disparity and incidences of COVID‐19 specifically in the African American population in US states followed by a focused study on 82 different counties in Mississippi. Our analysis revealed that in the US African Americans population are more prone to develop COVID‐19 infection compared to white Caucasian; we also found a correlation between occurrence of obesity and diabetes to COVID‐19 infection in African American population. In the state of Mississippi, African American population have an increased chance of getting COVID‐19 infection as a result of increased obesity rate and lower socioeconomic standard (lesser median income and lack of health insurance) compared to the white Caucasian population.

Mississippi has the maximum percentage of the African American population with an increased number of African American population being in poverty and without health insurance (https://www.census.gov/quickfacts/MS). Our analysis has revealed a positive correlation between lack of health insurance coverage to increasing COVID‐19 cases in the African American population. Health insurance will give the privilege to screen everyone for annual health check‐ups and assess health conditions. Several counties in Mississippi lack proper health access, which leads to poor health conditions in several areas. Health disparity has been a topic of discussion in the last few years due to a tremendous discrepancy of health‐related records between the races. It has been seen that hispanic and black populations are more prone to develop certain diseases such as cancer, diabetes, obesity, and cardiovascular diseases more than the white population.^29^, ^30^ This trend has been observed across the country with more prominence in the southern states of the United States. Human immunodeficiency virus infection, syphilis, chlamydiosis, obesity, cardiovascular disorders, and age‐adjusted mortality are specifically higher in the southern states in United States compared to other parts of the country.^31^, ^32^, ^33^ Poor nutrition and lack of health access both promote this condition in the minority population in the United States. A recent study by Goyal et al^34^ found that children from ethnic minority groups living in poor socioeconomic conditions are more prone to develop COVID‐19 disease more than groups with better socioeconomic status. This study found that children residing in household with low median family income have a higher incidence of COVID‐19 disease. Our analysis has also revealed a strong correlation between the poor economic condition and increased COVID‐19 disease in the African American population in Mississippi. Our study included data from all 82 counties of Mississippi. Association of comorbidities in the increasing incidents of COVID‐19 is observed during the pandemic in the United States; different demographics across the nation have found that comorbidities played an important role in the severity of COVID‐19 infection.^3^

Access to affordable healthcare in recent years has been instrumental in minimizing social and health disparities in the United States to some extent^35^ although the problems are still existing due to issues such as lack of health education, poor nutritional choices, and unemployment. A significant population in United States is still living without health insurance having no opportunity for annual health screening and monitoring https://www.census.gov/library/publications/2019/demo/p60-267.html). This population is already living in a vulnerable condition that could be worsened during the COVID‐19 pandemic.

Our findings necessarily point out the lack of health access and increasing pre‐existing conditions in the people of color in the State of Mississippi. Moreover, the insufficiency of proper nutritional choices is another contributing factor toward growing metabolic disorders and obesity.^36^ As a fact, Mississippi is ranked as the most obese state in the United States, which is a major concern as the population with extreme obesity are the ones having a compromised immune system as well. Huizinga et al^13^ have identified the impact of obesity induced dysregulation of the innate immune response, which triggers COVID‐19 infection and the severity of the disease.

Host genetic variations may also explain the variations in the susceptibility and severity of COVID‐19 found in different human populations. However, the present study is only emphasizing on the variants, which are crucial determinants of health disparity in context of COVID‐19 disease.

A solid and feasible health plan and options for regular health screenings are necessary to tackle situations like a pandemic in the future. Health education is one area that needs to be developed and expanded rapidly in the general population. Health access is poor in many different counties in Mississippi which is further making the condition severe and drastic during the COVID‐19 pandemic. An immediate action plan needs to be implemented in the poor health access areas to maintain a good health score of the residents. Preventive measures could be taken depending on the health score of the individuals; providing ideal nutritional choices is also required to maintain the good health of the community.

## CONFLICT OF INTEREST

The authors declare that there are no conflicts of interest.

## AUTHOR CONTRIBUTIONS

Conceptualization: Debarshi Roy, Sanjay Sarkar, Archie Taylor, Pratik Dutta

Data Collection: Praise Ola, Sofia Ievleva, Meghna Bajaj, Brenita Jenkins, Cardarius Llyod

Analysis and Interpretation of Data: Sanjay Sarkar, Debarshi Roy, Pratik Dutta, Bidisha Sengupta, Martha Ravola

Writing – Original Draft Preparation: Sanjay Sarkar, Archie Taylor, Debarshi Roy, Bidisha Sengupta

Writing – Review & Editing: Sanjay Sarkar, Archie Taylor, Debarshi Roy, Bidisha Sengupta, Martha Ravola, Brenita Jenkins, Justin Nash

Statistical Analysis: Sanjay Sarkar and Debarshi Roy

All authors have read and approved the final version of the manuscript.

Debarshi Roy had full access to all the data in this study and takes complete responsibility for the integrity of the data and the accuracy of the data analysis.

## TRANSPARENCY STATEMENT

Debarshi Roy affirms that this manuscript is an honest, accurate, and transparent account of the study being reported; that no important aspects of the study have been omitted; and that any discrepancies from the study as planned (and, if relevant, registered) have been explained.

## Data Availability

The data supporting the findings of this study are available from the corresponding author upon reasonable request.
